# Dynamic Transcriptome Profile Analysis of Mechanisms Related to Melanin Deposition in Chicken Muscle Development

**DOI:** 10.3390/ani14182702

**Published:** 2024-09-18

**Authors:** Gaige Ji, Ming Zhang, Xiaojun Ju, Yifan Liu, Yanju Shan, Yunjie Tu, Jianmin Zou, Jingting Shu, Hua Li, Weidong Zhao

**Affiliations:** 1 Key Laboratory for Poultry Genetics and Breeding of Jiangsu Province, Jiangsu Institute of Poultry Science, Yangzhou 225125, China; 2 School of Life Science and Engineering, Foshan University, Foshan 528231, China; 3 Taihe Fengsheng Agricultural and Livestock Co., Ltd., Ji’an 343732, China

**Keywords:** black-boned chicken, muscle, melanin deposition, DEGs, WGCNA

## Abstract

**Simple Summary:**

Darkness of the pectoral muscles is closely related to the amount of melanin deposited in the muscles of black-boned chickens. Embryonic development is an important stage in muscle melanin deposition. This study used RNA-seq to compare gene expression differences between the pectoral muscles of black-boned and non-black-boned chickens at different developmental periods. The results identified vital genes and EDN3/EDNRB2 signaling pathways involved in melanin deposition via GO and KEGG pathways and WGCNA analyses. Additionally, we identified five specific genes that may regulate melanin deposition in different breeds of black-boned chicken.

**Abstract:**

The pectoral muscle is an important component of skeletal muscle. The blackness of pectoral muscles can directly affect the economic value of black-boned chickens. Although the genes associated with melanogenesis in mammals and birds have been thoroughly investigated, only little is known about the key genes involved in muscle hyperpigmentation during embryonic development. Here, we analyzed melanin deposition patterns in the pectoral muscle of Yugan black-boned chickens and compared differentially expressed genes (DEGs) between the muscles of Wenchang (non-black-boned chickens) and Yugan black-boned chickens on embryonic days 9, 13, 17, and 21. Melanin pigments were found to gradually accumulate in the muscle fibers over time. Using RNA-seq, there were 40, 97, 169, and 94 genes were identified as DEGs, respectively, between Yugan black-boned chicken muscles and Wenchang chickens at embryonic day 9, 13, 17, and 21 stages (fold change ≥2.0, false discovery rate (FDR) < 0.05). Thirteen DEGs, such as *MSTRG.720*, *EDNRB2*, *TYRP1*, and *DCT*, were commonly identified among the time points observed. These DEGs were mainly involved in pigmentation, melanin biosynthetic and metabolic processes, and secondary metabolite biosynthetic processes. Pathway analysis of the DEGs revealed that they were mainly associated with melanogenesis and tyrosine metabolism. Moreover, weighted gene co-expression network analysis (WGCNA) was used to detect core modules and central genes related to melanogenesis in the muscles of black-boned chickens. A total of 24 modules were identified. Correlation analysis indicated that one of them (the orange module) was positively correlated with muscle pigmentation traits (r > 0.8 and *p* < 0.001). Correlations between gene expression and L* values of the breast muscle were investigated in Yugan and Taihe black-boned chickens after hatching. The results confirmed that *EDNRB2*, *GPNMB*, *TRPM1*, *TYR*, and *DCT* expression levels were significantly associated with L* values (*p* < 0.01) in black-boned chickens (*p* < 0.05). Our results suggest that *EDNRB2*, *GPNMB*, *TRPM1*, *TYR*, and *DCT* are the essential genes regulating melanin deposition in the breast muscle of black-boned chickens. *MSTRG.720* is a potential candidate gene involved in melanin deposition in the breast muscles of Yugan black-boned chickens.

## 1. Introduction

The black-boned chicken is an ancient breed known for its medicinal, meat, and ornamental value. The most obvious phenotypic difference between black-boned and non-black-boned chickens is the numerous amounts of melanin deposits in the body of the black-boned chicken. According to many studies, melanin has different physiological effects. Melanin eliminates free radicals in the body [1,2], improves the body’s immunity [3], exerts anti-cancer activity [4], inhibits viral infections [5], and exhibits anti-radiation effects [6], among others. Black-boned chicken breeds have become increasingly popular among consumers, owing to their meat and medicinal value. Based on previous observations, melanin deposition in the muscle changes dynamically. For example, the dark pigmentation of the muscle gradually becomes lighter as aging proceeds, indicating that the melatonin content gradually decreases. The blackness of the muscle is used by consumers to judge meat quality, and a high melanin content indicates a higher economic value. Thus, the level of muscle pigmentation is a key concern for breeders.

Melanin pigments are polymeric compounds synthesized by melanocytes from tyrosine, phenylalanine, and tryptophan and are deposited in melanosomes. Melanin is mainly found in two forms: eumelanin and pheomelanin. The color of eumelanin is black or dark brown, while that of pheomelanin is yellow or reddish-brown. The proportion of eumelanin plays a key role in determining the color phenotype of the skin, plumage, hair, and muscle in animals [7,8,9,10].

Multiple signaling pathways, including α-MSH/MC1R/cAMP [11], Wnt/β-catenin [12], SCF/c-Kit [13], GM-CSF [14], and MAPK [15,16] are involved in the regulation of melanogenesis. Several studies have identified candidate genes including *PMEL*, *GPR143*, *SLC24A5*, *GPNMB*, *MLPH*, *EDNRB2*, and *CALML4*, etc., which may play important roles in melanin deposition of chicken breast muscle using RNA sequencing (RNA-Seq) [17,18]. Genes such as *MYL1*, *RPS14*, *MYOZ2*, and *MYOD1*, which are associated with muscle growth were also found to contribute to melanogenesis in the breast muscle of black-boned chicken [19]. Most of the above studies have focused on the adult stage of breast muscle by comparing the gene expression differences between hyperpigmentation and hypopigmentation in black-boned chicken. However, according to our observations, the embryonic stage is an important stage for muscle melanin deposition in chicken. After hatching, the breast muscle blackness gradually decreases with increasing age. Despite current knowledge of the genes and signaling pathways involved in hyperpigmentation, the expression patterns of key genes in chicken meat pigmentation are still poorly understood, especially those of genes in embryonic development. Yugan black-boned chicken is a native chicken breed to Yugan County in the Jiangxi province of China and is characterized by its black skin, feathers, beaks, crowns, flesh, periosteum, toes, muscle, and visceral organs, which were selected as sample materials in this study. Wenchang chickens (non-black-boned chickens), which have similar body weight to that of the newly hatched offspring of Yugan black-boned chickens, were employed as a control in this study. The objectives of this study were to collect the pectoral muscles of Wenchang and Yugan black-boned chickens and perform dynamic transcriptome profiling to identify the key genes involved in muscle hyperpigmentation. The findings of this study improved our understanding of the molecular mechanisms of muscle hyperpigmentation and molecular breeding related to muscle melanin deposition in black-boned chickens.

## 2. Materials and Methods

### 2.1. Animals and Tissue Collection

Fertile eggs of Wenchang and Yugan black-boned chickens were purchased from the National Grade Breeding Farm. The two varieties of fertile eggs were incubated in an incubator using conventional methods. Three embryos from each variety were randomly selected every day from day 5 to 21 for observation. Of note, melanin began to appear in the pectoral muscle of Yugan black-boned chickens on embryonic day nine (E9). Thereafter, the black color of the pectoral muscle gradually deepened. Finally, six embryos of Yugan black-boned chickens were randomly selected at embryonic days 9 (E9), 13 (E13), 17 (E17), and 21 (E21). Three embryos were used to obtain pectoral muscle samples for total RNA extraction, and the other three embryos were used to obtain pectoral muscles for the preparation of paraffin tissue sections on incubation days 9, 13, 17, and 21. During the same observation period, three Wenchang chicken embryos were randomly selected, and the pectoral muscle was sampled for total RNA extraction to serve as the control. 

In this study, six hens were selected for sample collection, including Yugan black-boned chicken and Taihe black-boned silky fowl from each time point after hatching. All experimental chickens were raised under the same conditions and slaughtered at the age of 2, 6, 10, 14, and 16 weeks (W). The L* (light to dark axis) values of pectoral muscle were measured using an NR20XE colorimeter (3NH, Guangzhou, China). The left muscle was obtained after dissection and was designated for RNA extraction. The right muscle at the same position was sampled for histological examination.

Muscle samples were fixed in a 10% formaldehyde solution, embedded in paraffin, mounted onto slides, and stained with Masson–Fontana stain according to the manufacturer’s instructions (SolarBio, Beijing, China).

### 2.2. Total RNA Extraction and Library Preparation for Sequencing

Total RNA from the pectoral muscles of chicken embryos on E9, E13, E17, and E21 was extracted using TRIzol Reagent (Invitrogen, Carlsbad, CA, USA), according to the manufacturer’s protocol. RNase-free agarose gel electrophoresis and Agilent 2100 Bioanalyzer (Agilent Technologies, Palo Alto, CA, USA) were used to assess RNA quality. The isolated RNA was then enriched with oligo(dT) beads and fragmented using a fragmentation buffer. First-strand cDNA synthesis was subsequently performed using random primers. Second-strand cDNA was synthesized using DNA polymerase I, RNase H, dNTP, and a buffer. The cDNA fragments were purified using a QiaQuick PCR extraction kit (Qiagen, Venlo, The Netherlands) and end-repaired. Poly(A) tails were then added and ligated to Illumina sequencing adapters. The ligation products were size-selected via agarose gel electrophoresis, amplified via PCR, and sequenced using an Illumina HiSeq2500 by Gene Denovo Biotechnology Co. (Guangzhou, China).

High-quality clean reads were further filtered from raw reads using fastp version 0.18.0 [20]. An index of the reference genome was constructed, and paired-end clean reads were mapped to the reference genome (Galgal 6.0) using HISAT2. 2.4 [21], with “RNA-strandness RF” and other parameters set as the default. 

### 2.3. Analysis of Differentially Expressed Genes (DEGs)

Mapped reads of each sample were assembled using StringTie v1.3.1 [22,23] and a reference-based approach. The fragment per kilobase of transcript per million mapped reads (FPKM) for each transcription region was calculated to quantify its expression abundance and variations using the StringTie software (https://ccb.jhu.edu/software/stringtie/). RNA differential expression analysis between the two groups was performed using DESeq2 (1.20.0) [24] software. Genes with a false discovery rate (FDR) < 0.05 and absolute fold-change (FC) ≥ 2 were considered DEGs.

Gene Ontology (GO) enrichment analyses of the DEGs were conducted using the Gene Ontology database (http://www.geneontology.org/, accessed on 1 September 2020). Pathway enrichment analysis was performed using the KEGG database. The significance level for the GO terms and KEGG pathways was an FDR or *p* < 0.05. 

### 2.4. Weighted Gene Co-Expression Network Analysis (WGCNA)

Wenchang and Yugan black-boned chicken muscle samples were used to construct an unsigned co-expression network. Genes with FPKM values < 1 in all samples were removed prior to analysis. The pickSoftThreashold was used to calculate the appropriate soft threshold β. The topological overlap matrix (TOM) was used to construct the WGCNA network, with the following parameters: soft threshold (β), 6; minimum module size, 30; and MergeCutHeight, 0.25. Based on the changes in pectoral muscle color, four gradients were set for the corresponding time points when the Yugan black-boned chicken pectoral muscles were observed (E9 = 1, E13 = 2, E17 = 3, E21 = 4). The blackness of the pectoral muscle of Wenchang chickens was set to zero at all time points. Thereafter, the modules that were highly correlated with changes in pectoral muscle pigmentation could be identified. The modules with the highest correlation coefficient among all modules correlated with the variation in blackness (positively or negatively) were selected for further analyses.

### 2.5. mRNA Expression Analyses via Quantitative PCR (qPCR)

Six muscle samples from each breed were used to detect the expression levels of target genes at each time point. Total RNA (1 μg) from each sample was extracted and transcribed into cDNA using an RNA-easy isolation reagent (Vazyme, Nanjing, China) and HiScript^®^ III-RT SuperMix for qPCR (+gDNA wiper) (Vazyme), according to the manufacturer’s instructions. The expression levels of the DEGs were quantified via qPCR using the ChamQ Universal SYBR qPCR Master Mix. The primer sequences used for qPCR are listed in Table S1. qPCR was performed using a Max3000P qRT-PCR system (Agilent, Santa Clara, CA, USA). Gene expression was normalized using the 2^−ΔΔCT^ method, with β-actin as the internal standard. The Yugan muscle samples of the two-week-old group were selected as the control group to detect the relative expression levels of target genes in other groups.

### 2.6. Statistical Analysis

Bioinformatics analysis was performed using OmicSmart, a real-time interactive online platform for data analysis (http://www.omicsmart.com, accessed on 1 September 2020). Data are expressed as mean ± standard error (SE). The significance of the difference between two groups at the same time point was determined using an independent samples *t*-test in the SPSS software (SPSS version 20.0). The expression difference in the genes was statistically compared among the different time points using one-way analysis of variance (ANOVA) followed by the Turkey test, as implemented in the SPSS Software Suite. Correlation analysis was conducted by Pearson correlation. Differences were considered statistically significant at *p*-values <0.05 and <0.01.

## 3. Results

### 3.1. Histological Observation of the Changes in Melanin Production between Wenchang and Yugan Black-Boned Chickens at Different Development Stages

**Figure 1:**
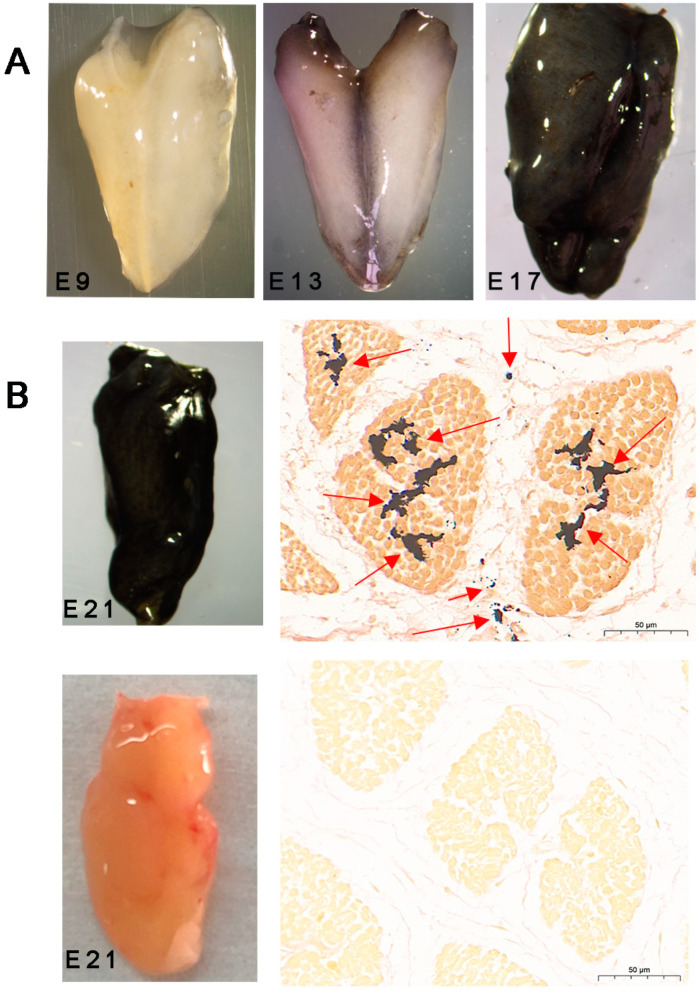
Morphological and histological micrographs of the pectoral muscles of Yugan black-boned chickens at different stages of embryonic development. (**A**) Morphological photographs of Yugan black-boned chicken muscle from E9 to E17. (**B**) Muscle morphological and corresponding histological photographs of Yugan (first row) black-boned chicken and Wenchang (second row) chicken at E21. E9: embryonic day 9. E13: embryonic day 13. E17: embryonic day 17. E21: embryonic day 21. Arrows: melanin particles.

Obvious color differences were not found between the pectoral muscles of Wenchang and Yugan black-boned chickens until E9. At E9, the pectoral muscles of the Yugan black-boned chicken attached to the bone appeared as black dots (Figure 1A). The color of the pectoral muscles attached to the bone became darker, while the middle part of the pectoral muscles remained white. Thereafter, the black part of the muscle increased, and from E13 to E17, the color became increasingly darker. At E21, the entire pectoral muscle of Yugan black-boned chickens turned black, and the pectoral muscle retained a normal light-red color in Wenchang chickens. Using the Masson–Fontana staining method, melanin in the muscle tissues was stained black with a yellow background. The results showed multiple large spots of melanin deposited in the muscle fibers and peripheral membranes (Figure 1B). No obvious melanin granules were found in the Wenchang chicken muscles at E21.

### 3.2. Differential Expression Analysis during Muscle Development

The muscles of Wenchang (W) and Yugan black-boned (Y) chickens were sampled at E9, E13, E17, and E21, with three biological replicates for RNA sequencing analysis. Twenty-four cDNA libraries were constructed based on the pectoral muscle of Wenchang and Yugan black-boned chickens collected at different stages of development. A total of 938,440,164 raw reads were obtained, and 934,739,458 clean reads were acquired after filtering and pruning. The Q20 and Q30 values were >97 and >92%, respectively, for the 24 samples, that met the requirements of the follow-up analysis. The total reads mapped to the chicken genome for these samples ranged from 82.97% to 90.66%, with 1.69% to 2.64% multiple mapped reads, and 80.94% to 88.74% uniquely mapped reads (Table 1).

**Figure 2:**
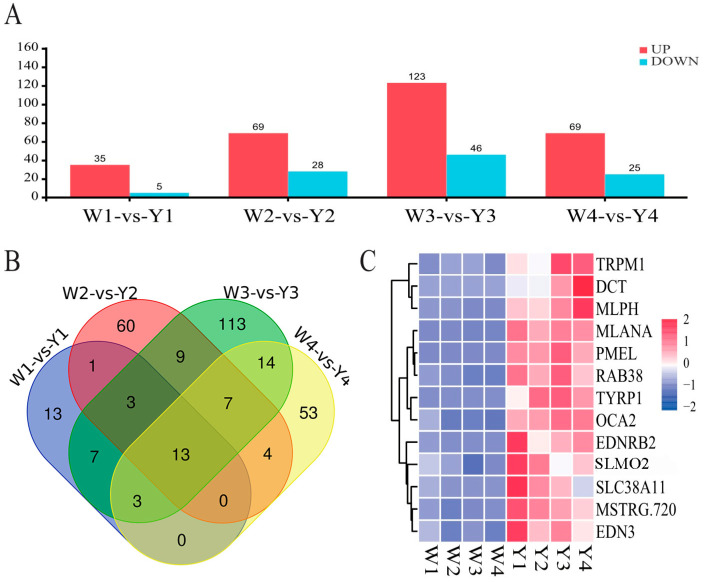
Analysis of differentially expressed genes (DEGs). (**A**) DEGs statistics between Wenchang (W) and Yugan (Y) black-boned chicken at different time points. (**B**) The Venn analysis of DEGs. (**C**) Clustering heatmap of 13 common DEGs. The blue to red color displays low to high expression levels, respectively. W1/2/3/4: Wenchang chickens at embryonic days 9, 13, 17, and 21, respectively. Y1/2/3/4: Yugan chickens at embryonic days 9, 13, 17, and 21, respectively. The same as below.

The DEGs in the four developmental stages were analyzed, and 40, 97, 169, and 94 DEGs were identified on E9, E13, E17, and E21, respectively (|FC| > 2, *FDR* < 0.05) (Figure 2A). Detailed information on the DEGs was listed in Table S2. Genes such as *EDN3*, *GPNMB*, *RAB38*, *OCA2*, *TRPM1*, *MLPH*, *TYRP1*, *PMEL*, and *DCT* that are associated with pigmentation were identified. The genes related to adhesion molecules, lipid metabolism, skeletal system development, and bone development such as *COL6A3*, *TNN*, *TNX*, *MYH1E*, *PLIN1*, *FABP4*, and *SHH* were also identified. At the same time, a series of novel genes were discovered as DEGs between two breeds at different time points. The expression levels of the *MSTRG.720* were increased by about 2-fold in Yugan black-boned chicken at the four-time points. The *MSTRG.17193* and *MSTRG.17192* expression levels were upregulated by more than 10-fold in Yugan black-boned chicken at E9 and E17. The *MSTRG.18980* expression levels were upregulated by more than 8-fold in Yugan black-boned chicken at E9, E13, and E21 (Table S2).

**Figure 3:**
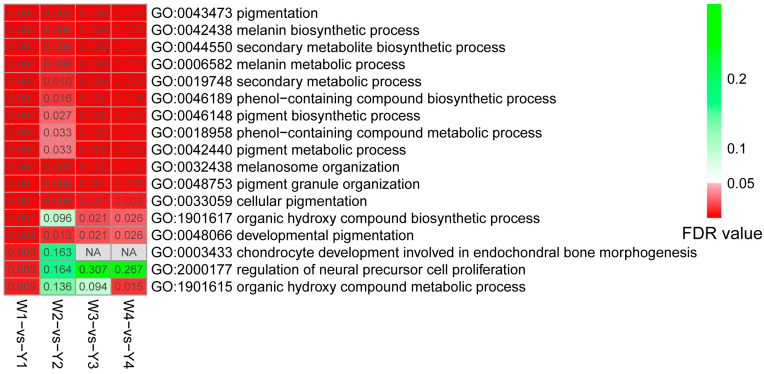
Gene ontology enrichment of the biological process terms for DEGs identified between the Wenchang and Yugan black-boned chicken muscle groups at different developmental stages. Significantly enriched GO terms in the four groups are used (FDR values < 0.05). Significant FDR-values are indicated in red, whereas non-significant FDR values are indicated in green. NA represents the corresponding terms that are not enriched.

Thirteen common DEGs were identified from the four developmental stages using Venn analysis (Figure 2B). The 13 genes (*MLANA*, *EDN3*, *RAB38*, *OCA2*, *TRPM1*, *SLC38A11*, *MLPH*, *SLMO2*, *PMEL*, *TYRP1*, *DCT*, *EDNRB2*, and *MSTRG.720*) all showed upregulated in muscles of Yugan black-boned chickens when compared with Wenchang chickens (Figure 2C). GO enrichment analysis was performed to functionally characterize the DEGs (Figure 3 and Table S3). Based on this analysis, the significantly enriched biological processes across development stages included pigmentation, melanin biosynthetic and metabolic processes, secondary metabolite biosynthetic process, phenol-containing compound biosynthetic process, cellular pigmentation, and pigment granule organization. The chondrocyte development involved in endochondral bone morphogenesis (GO:0003433) and regulation of neural precursor cell proliferation (GO:2000177) terms were only significantly enriched in the E9 stage. Three genes *SHH*, *MATN1*, and *DCT* were involved in the two biosynthetic processes. The organic hydroxy compound metabolic process (GO:1901615) was significantly enriched in the E9 and E21 stages.

**Figure 4:**
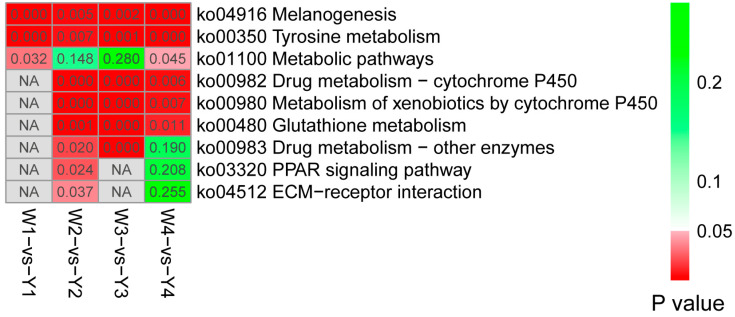
KEGG pathway analysis of the DEGs between the Wenchang and Yugan black-boned chicken embryo muscle groups at different developmental stages. Significantly enriched KEGG pathways in the four groups are used (*p* values < 0.05). Significant *p*-values are indicated in red, whereas non-significant *p*-values are indicated in green. NA represents the corresponding pathways that are not enriched.

The KEGG pathway analysis of the DEGs revealed that melanogenesis, tyrosine metabolism, and metabolic pathways were common and significantly enriched across the four developmental stages (Figure 4 and Table S4). The drug metabolism–cytochrome P450 (ko00982), metabolism of xenobiotics by cytochrome P450 (ko00980), and glutathione metabolism (ko00480) pathways were common and significantly enriched in E13, E17, and E21 stages. *GSTO2*, *HPGDS*, *LOC100857615*, *GSTA2*, *GSTA3*, and *GSTT1L* genes were involved in these pathways. The drug metabolism–other enzymes (ko00983) pathway was significantly enriched in the E13 and E17 stages. DEGs *GSTO2*, *GSTA2*, *GSTA3*, and *LOC100859645* were involved in the pathways. The PPAR signaling pathway (ko03320) (including *PLIN1* and *FABP4*) and ECM–receptor interaction pathway (ko04512) (including *COL6A3* and *TNX*) were both only significantly enriched in the E13 stages.

### 3.3. WGCNA

**Figure 5:**
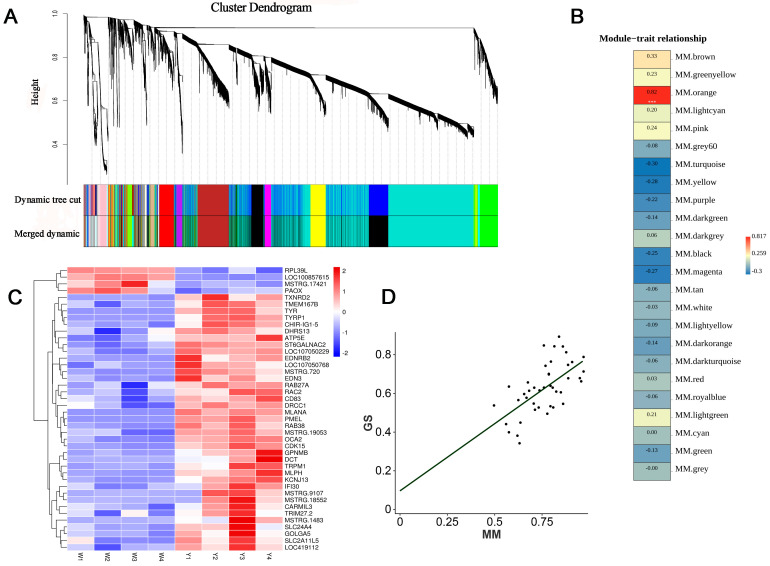
Gene modules based on weighted gene co-expression network analysis (WGCNA). (**A**) Identification of the co-expression modules and cluster dendrogram. **Upper panel**: genes clustered into different groups. **Lower panel**: genes assigned to network modules after dynamic tree cut and merging analyses. Various colors represent different modules. (**B**) Relationships between the orange module and muscle pigmentation traits. Each line presents the module, and each block contains the correlation coefficient and *p*-value (*** represents *p* < 0.001). (**C**) Clustering heat map of genes in the orange module. Blue to red color displays low to high expression levels, respectively. (**D**) Correlation analysis results of the orange module between gene significance (GS) and module membership (MM).

After excluding genes with FPKM values < 1, 14,449 genes were included in the WGCNA. Twenty-four gene modules were identified, with a power value of six (Figure 5A). The orange module was strongly and positively correlated with muscle pigmentation (r > 0.8 and *p* < 0.001) (Figure 5B and Table S5). Correlation analysis revealed a significant positive correlation between gene significance (GS) and module membership (MM) (r = 0.62 and *p* < 0.001) (Figure 5D), indicating that the 42 genes in the orange module were highly correlated with melanin deposition. Most of the genes in the orange module showed significantly upregulated expression in the black-boned chicken muscle (Figure 5C). The expression levels of *ST6GALNAC2*, *LOC107050229*, *EDNRB2*, *LOC107050768*, *MSTRG.720*, and *EDN3* mRNA showed a peak at E9. *TMEM167B*, *TYR*, *TYRP1*, *CHIR-IG1-5*, and *DHRS13* mRNA showed two peaks at E13 and E17. *CDK15*, *TRPM1*, *IFI30*, *MSTRG.18552*, *CARMIL3*, *TRIM27.2*, *MSTRG.1483*, *SLC24A4*, *GOLGA5*, *SLC2A11L5*, and *LOC419112* mRNA showed a peak at E17. *ATP5E*, *GPNMB*, *DCT*, *MLPH*, *CD83*, and *KCNJ13* mRNA showed a peak at E21. Genes *RPL39L*, *LOC100857615*, *MSTRG.17421*, *PAOX* showed significantly upregulated expression in Wenchang chicken muscles. GO enrichment analysis of the orange module was also performed (Table S6). The biological process terms were found to be mainly significantly enriched in pigmentation, melanin, and secondary metabolite biosynthetic processes, phenol-containing compound metabolic processes, cellular pigmentation, pigment granule organization, and organic hydroxy compound biosynthetic processes, and related metabolic processes. Further, the molecular function terms were significantly enriched in monooxygenase activity, oxidoreductase activity, monophenol monooxygenase activity, oxygen atoms, and copper ion binding. The cell component terms were significantly enriched in melanosomes, pigment granules, melanosome membranes, chitosomes, cytoplasmic vesicles, integral components of membranes, and melanosome lumens. KEGG results revealed that four pathways were significantly enriched, including melanogenesis, tyrosine metabolism, selenocompound metabolism, and other glycan degradation pathways (Table S7). *EDNRB2* is a paralog of EDNRB (the endothelin B receptor, ETB-R). The four genes, *EDNRB2*, *TYRP1*, *TYR*, and *DCT* were included in the melanogenes and tyrosine metabolism signaling pathways.

### 3.4. Analysis of Core Pathways and Genes Associated with Muscle Melanin Deposition

**Figure 6:**
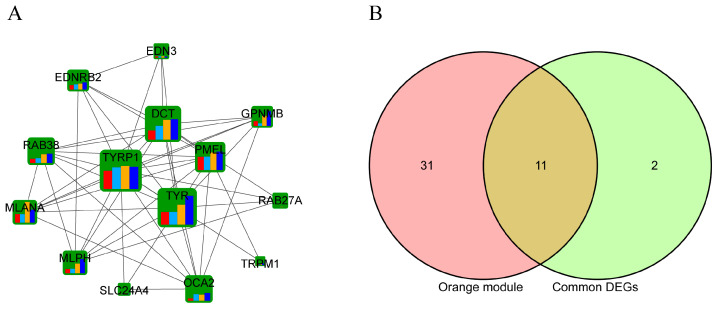
Screening of the hub genes. (**A**) Protein–protein interaction (PPI) network of genes in the orange module. Bars in the nodes represent the logFC value detected at different time points (red: E7; light blue: E13; yellow: E17; heavy blue: E21). The sizes of the nodes indicate the degrees to which the nodes connect to others. (**B**) Venn analysis between the orange module and common DEGs found between Wenchang and Yugan black-boned chicken muscle samples at different developmental stages.

A gene co-expression network was constructed in the orange module to identify the hub genes. The network was constructed using the STRING database and visualized using Cytoscape software (https://cytoscape.org/). The hub genes identified in the orange module following the removal of the branch network are shown in Figure 6A. The hub genes, *TYRP1*, *TYR*, *DCT*, and *PMEL*, showed higher degrees than other genes in the network. A Venn analysis was performed between the orange module and the common DEGs found at the four developmental stages. Eleven genes (*MSTRG.720*, *EDNRB2*, *DCT*, *TYRP1*, *PMEL*, *MLPH*, *TRPM1*, *OCA2*, *RAB38*, *EDN3*, and *MLANA*) were common in the Venn analysis (Figure 6B). The expression levels of the DEGs, *MSTRG.720*, *EDNRB2*, *DCT*, *TYRP1*, and *EDN3*, and the non-DEG, *MITF*, in the six collected samples, were determined using qPCR. There was a high positive correlation (R^2^ = 0.803) between the RNA-seq and qPCR fold changes results (Figure S1). 

### 3.5. Correlation Analysis between Gene Expression and L* Values of Muscle Darkness in Different Breeds of Black-Boned Chickens

**Figure 7:**
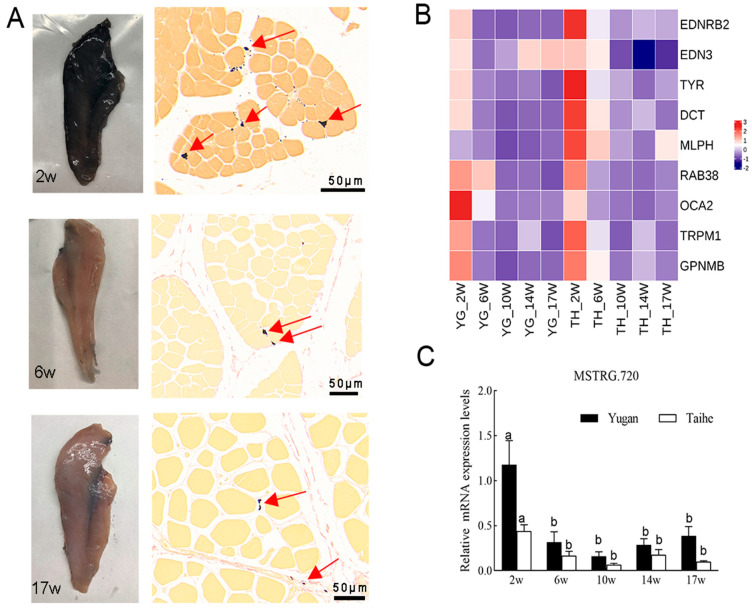
(**A**) Morphological and corresponding histological micrographs of the pectoral muscles of Yugan black-boned chickens at 2 (2 W), 6 (6 W), and 17 weeks (17 W) of age after hatching. (**B**) The expression changes of the nine genes in Yugan (YG) and Taihe (TH) black-boned chicken muscle groups at different developmental stages. (**C**) Expression of *MSTRG.720* in Yugan (YG) and Taihe (TH) black-boned chicken muscle groups at different developmental stages. Data with different letters on each bar mean statistically significant among different time points for the same breeds (*p* < 0.05). Arrows: Melanin particles.

As illustrated in Figure 7A, muscle darkness gradually decreased with age in Yugan black-boned chickens. Similar patterns were observed in Taihe black-boned chickens (Figure S2). Histologic testing showed a reduction in melanin content in Yugan chicken muscle from two weeks (2 W) to six weeks (6 W) post-hatching. Melanin spots in the pectoral muscle were barely located in the muscle fibers and were mainly distributed in the blood vessels and surrounding membrane tissue. The L* values of muscle darkness in the two black-boned chickens were the lowest value at 2 W, increasing significantly (*p* < 0.05) from 6 W to 17 W (Figure S3). 

To further detect the key candidate genes affecting muscle darkness, the expression levels of nine genes in the orange module were quantified in different chicken breeds using qPCR (Figure 7B). *MSTRG.720* showed high mRNA gene expression in the 2 W but significantly decreased at 6 W in Yugan black-boned chicken muscles (*p* < 0.05). After 6 W, the *MSTRG.720* expression level remained relatively stable until 17 W (Figure 7C). Other genes (*EDNRB2*, *TYR*, *DCT*, *OCA2*, *TRPM1*, and *GPNMB*) showed high expression levels at the 2 W stage, significantly decreased at 6 W (*p* < 0.05), and remained at a relatively stable level until 17 W. Similar change trends in these genes were observed in Taihe (TH) black-boned chicken muscles. The expression level of *EDN3* was upregulated at 14 W and 17 W, and there was no significant difference among the different time points in Yugan black-boned chicken muscles (*p >* 0.05). *MLPH* expression was significantly increased in Taihe black-boned silky fowl at 17 W age (*p* < 0.05). *RAB38* expression remained relatively high from 2 W to 6 W, significantly downregulated in Yugan black-boned chicken muscles (*p* < 0.05).

The expression of the *MSTRG.720* gene was significantly negatively correlated (r^2^ = −0.486, *p* < 0.05) with the L* values of muscle darkness in Yugan black-boned chickens but did not reach a significant level in Taihe black-boned silky fowl (r^2^ = −0.337, *p* = 0.086). The expression of *EDNRB2*, *TRPM1*, *GPNMB*, *TYR*, and *DCT* significantly negatively correlated (*p* < 0.05) with L* values of muscle darkness in Yugan and Taihe black-boned chickens (Table 2). EDN3, RAB38, OCA2, and MLPH expression were significantly negatively correlated (*p* < 0.05) with L* values of muscle darkness in Taihe black-boned chickens.

## 4. Discussion

The amount of melanin deposited in the tissues and organs of black-boned chickens varies across the developmental stages [25], suggesting that melanin accumulation may be controlled by multiple genes. The pectoral muscle is an important meat-producing part of black-boned chickens, and melanin deposition in the muscle can directly affect the economic value of these birds. According to our preliminary observations, the embryonic stage is critical for muscle pigmentation. Wenchang chicken and Yugan black-boned chicken belong to different branches in the phylogenetic consensus tree and have a distant genetic distance [26]. Thus, we selected the pectoral muscles of Wenchang and Yugan black-boned chickens at different developmental stages to analyze the core genes or pathways that control melanin deposition in the muscles.

An important difference between regular and black-boned chickens is the absence of excessive melanin deposition in the body tissues. As expected, melanin was barely visible in the muscles of Wenchang chickens at both the embryonic and adult stages (Figure S4). The L* value represents the lightness and darkness (white and black, respectively), with larger values indicating whiter (brighter) colors and lower melanin content. After six weeks of age, the L* values of the breast muscle in the two black-boned chickens increased significantly, indicating that muscle coloration became brighter. At 17 weeks of age, the L* value was significantly lower than that of Wenchang chickens (Table S9), indicating that the muscle color of black-boned chickens was darker than that of Wenchang chickens.

Melanin deposition in the muscle of Yugan black-boned chickens is dynamic from the embryonic stage to adulthood (Figure 1 and Figure 7). Embryonic age is a key stage at which melanin deposition gradually increases. After hatching, the melanin content of muscles gradually decreases with age. These findings align with those of previous research on the skin [27]. Nishimura et al. [28] found that this phenomenon may be linked to variations in melanocyte numbers across different age groups. Studies on human skin and hair have suggested a correlation between melanocyte proliferation and hormones in the developmental processes. However, the precise regulatory mechanisms remain unclear [29,30,31]. Hence, differences in melanin content across varying ages in muscles may be related to growth factors and development-related hormones. Histological analysis revealed that the distribution of melanin at the embryonic stage differed from adult chicken muscles. Shi et al. [32] concluded that the deposition and disappearance of melanin in the pectoral muscle followed a certain order. We speculated that melanin granules accumulated in muscle fibers may have been the first to be lost in adult black-boned chickens. 

Melanin synthesis mainly occurs in melanocytes that originate from the neural crest (NC) and then proliferate and migrate to the eye uvea, skin, and muscle as the embryo develops [33,34]. Melanosomes are specialized organelles in melanocytes responsible for melanin synthesis, storage, and transport. Mature melanosomes accumulate in melanocyte dendritic structures and are transported to keratinocytes and neighboring cells via cell phagocytosis, or membrane fusion, consequently coloring the skin or hair [35]. Melanin deposition in muscle tissue may be similar to that in the skin or hair. In this study, melanin was observed along the sternum, spreading to the surrounding fascial tissue and muscles on both sides as time progressed. Melanosomes are transferred from melanocytes through intercellular phagocytosis, membrane fusion, and direct transfer to muscle fibers and neighboring cells. The cytoskeletal components, microtubules, and microfilaments are also involved in intracellular pigment organelle transport [36].

The diversity of avian feather colors was found to be controlled by the distribution or differentiation of melanocytes [37,38]. The number and distribution of melanocytes determine the amount of melanin deposition in the animal body to a certain extent. Thus, melanocyte proliferation, differentiation, and migration are important for melanin deposition in animal tissues. By comparing the DEGs between the breeds, most DEGs were found to be upregulated in the muscles of Yugan black-boned chickens compared to those of Wenchang chickens. It can be hypothesized that most of these upregulated gene expressions were associated with increased melanin synthesis in black-boned chickens. GO enrichment confirmed that multiple terms related to melanin biosynthesis and metabolic processes were significantly enriched (FDR < 0.05). The KEGG analysis showed *EDN3* and *EDNRB2* signaling pathway was significantly enriched at the time points evaluated in the present study. The expression peaks of these two genes emerged at E9 when black dots began to appear in the muscle. Increased expression of *EDN3* can induce the proliferation and self-renewal of NC-derived melanocyte precursors in avian embryos [39,40]. *EDNRB2*, which is involved in melanoblast differentiation and migration along the dorsolateral pathway, has been identified as a key gene in the regulation of melanin deposition in the muscle of adult black-boned chicken [41,42,43]. Multiple signaling pathways, including calcium mobilization, MAPK/ERK, PKC, and cAMP/PKA pathways, could be activated by the EDN3/EDNRB pathway [44,45,46]. Additionally, reports have indicated that the melanin content deposited in the muscle is influenced by the muscle fiber composition. Melanin particles have a propensity to accumulate in athletic muscles [32]. Compared to other tissues, muscles are mainly composed of muscle fibers. Ca^2+^ influx from the sarcoplasmic reticulum triggers muscle contraction, leading to pigment particle accumulation. The calcium and MAPK signaling pathways were enriched in the orange module based on the WGCNA results. These two pathways may be regulated by the EDN3/EDNRB2 pathway. The EDN3/EDNRB2 pathway may regulate these two pathways to promote melanocyte proliferation, differentiation, and migration and then accelerate melanin deposition in the embryonic pectoral muscles.

MITF, a transcription factor, controls multiple signal transduction pathways and key genes, such as *TYR*, *TYRP1*, and *PMEL*, which are involved in melanogenesis [47]. The results showed no significant differences in *MITF* gene expression between the pectoral muscles of black-boned chickens and non-black-boned chickens. Similar results were found in other studies on melanin deposition in the muscle and skin of adult chickens and sheep [48,49,50]. It seemed that *MITF* was required but was not sufficient to induce the expression of the melanogenic gene. Gaggioli et al. [51] suggested that there may be unknown mechanisms that cooperate or synergize with *MITF* to regulate the expression of melanin-related genes and melanin synthesis. On the other hand, previous studies indicated that multiple splice variants of the *MITF* gene regulate the skin and hair pigmentation in sheep and buffalo [52,53]. There may be another alternative splicing of the *MITF* gene that regulates muscle melanin deposition in birds. Our findings provide new clues for investigating the molecular mechanisms of melanin production in the muscles of black-boned chickens. Melanocortin 1 receptor (*MC1R*) and agouti signaling protein (*ASIP*) genes were reported to control pigmentation in chicken skin or feather color [27,54,55]. The 2 genes (*MC1R* and *ASIP*) showed no significant difference in the present results indicating that there are differences in the molecular regulating mechanisms of melanin deposition between muscle and skin (or feather follicle) tissues.

Results from different black-boned chicken breeds identified *ANKRD1*, *MYOZ2*, and *MYOD1* genes associated with skeletal muscle development identified as hub genes playing essential roles in skeletal muscle melanogenesis [19]. Seven genes *PMEL*, *RAB29*, *SLC6A9*, *SLC38A4*, *SLC22A5*, *SLC35F3*, and *SLC16A3* have been identified as candidate genes that regulate melanin deposition in the breast muscle of Muchuan black-boned chickens [48]. The expression levels of these genes showed no significant differences between breeds in the present study, except for *PMEL*. Therefore, we hypothesized that differences in the genetic backgrounds of black-boned chickens might affect gene expression, regulating melanin production in the breast muscles of different black-boned chicken breeds.

Based on the WGCNA results, nine hub genes in the orange module were selected to investigate whether their expression was related to the L* value of breast muscle in different breeds of black-boned chickens after hatching. *Rab38* regulates tyrosinase vesicle transport from the Golgi to the melanosomes in mouse melanocytes [56,57]. *MLPH* is a significant component of melanosome transport via myosin [58]. *OCA2* is thought to regulate melanosomal pH, affecting *TYR* activity and melanin synthesis [59]. All these genes are necessary for melanogenesis. The results showed that EDN3, RAB38, OCA2, and MLPH gene expression levels significantly correlated with L* values in the breast muscle of Taihe black-boned silky fowl after hatching. Yugan black-boned chicken is genetically distant from Taihe silky fowl and belongs to different taxa [60]. Therefore, breed and age differences may affect the expression of genes associated with muscle pigmentation. 

The expression levels of *EDNRB2*, *GPNMB*, *TRPM1*, *DCT*, and *TYR* were significantly correlated with L* values in the breast muscle of the two black-boned chicken breeds. *TRPM1*, as a calcium channel, is critical for normal melanocyte pigmentation in mammalian skin tissue [61,62]. *PMEL* is a melanocyte-specific type I transmembrane glycoprotein responsible for fibril formation in premelanosomes and is involved in melanosome maturation [63,64]. The *GPNMB* protein is highly homologous to *PMEL* but has disparate functions in melanosome biogenesis. The overexpression of *GPNMB* can promote melanin deposition in chicken melanocytes [65]. TYR, TYRP1, and DCT are vital enzymes that regulate the production of pigments because of their essential roles in the final step of melanin synthesis [66,67]. It can be concluded that increased *EDNRB2* gene expression facilitates calcium mobilization and subsequently enhances *TRPM1* expression. This, in turn, leads to increased enzymatic activity of TYR and DCT, upregulation of GPNMB expression, and accelerated melanin deposition in chicken melanocytes. Notably, mRNA expression levels of *EDNRB2*, *DCT*, and *TYRP1* in Wenchang chicken muscle were minimally detectable, suggesting an association between the upregulated expression of these genes and excessive melanin synthesis and deposition in the breast muscle of the black-boned chicken.

In addition to the known genes, we identified multiple new or unfamiliar genes involved in melanogenesis, including *MSTRG.720*, *SLMO2*, *MSTRG.18980*, *ST6GALNAC2*, *DRCC1*, *LOC107050229*, and *SLC38A11.* The present results showed that *MSTRG.720* expression was significantly positively correlated with L* values in the breast muscle of Yugan black-boned chickens after hatching. The expression of *MSTRG.720* also exhibited a significant positive correlation with *EDNRB2*, *RAB38*, *OCA2*, *GPNMB*, *TRPM1*, *MLPH*, and DCT in the two black-boned chickens (Table S8). Homology analysis revealed that the MSTRG.720 sequence was 99.4% identical to the ncRNA (XR_005845017). Li reported that *EDN3* may regulate melanin production in black skin through interaction with the ncRNA LOC101747896 in black-bonded chickens [68]. *TYR* and *DCT* are target genes of several long non-coding RNAs in breast muscle and skin melanin deposition in black-boned chickens [69,70]. We speculate that *MSTRG.720* acts as a non-coding RNA involved in melanin deposition and reduction in the breast muscles of Yugan black bone chickens. Previous research has shown that *SLMO2* is located near EDN3 and ATP5e and is involved in skin melanin production [68]. However, little is known about the mechanisms underlying the interactions between *SLMO2* and other genes. Numerous genes associated with melanogenesis have been identified using high-throughput sequencing technology [19,48,49]; however, the interactions or regulatory mechanisms among these genes remain unclear in chickens. These newly discovered genes may help us more deeply analyze the regulatory mechanisms of muscle melanogenesis in chickens.

## 5. Conclusions

In summary, the present study used RNA-Seq to explore key genes and pathways underlying embryonic development that related to melanin deposition in the breast muscle of Yugan black-boned chicken. RNA-Seq results found genes in the orange module were crucial for melanogenesis in the breast muscle of black-boned chickens. Meanwhile, correlation results between gene expression and muscle L*-value confirmed that *EDNRB2*, *GPNMB*, *TRPM1*, *TYR*, and *DCT* were identified as the most potential candidate genes involved in the regulation of melanin deposition and muscle darkness in different breeds of black-boned chickens. This study provides a theoretical foundation for further understanding the regulatory mechanisms of breast muscle darkness in black-boned chickens.

## Figures and Tables

**Table 1:** Statistics and mapping results of the RNA-seq data for 24 samples.

Groups	Sample	Raw Reads	Clean Reads	Q20 (%)	Q30 (%)	Mapped on Reference	Unmapped	Uniquely Mapped
W1 (E9)	W1-1	38,053,396	37,885,648 (99.56%)	97.70%	93.54%	33,155,987 (87.73%)	4,638,405 (12.27%)	32,517,329 (86.04%)
	W1-2	41,571,114	41,415,818 (99.63%)	97.81%	93.77%	36,355,816 (87.96%)	4,976,584 (12.04%)	35,655,625 (86.27%)
	W1-3	39,111,530	38,933,226 (99.54%)	97.63%	93.38%	33,678,652 (86.69%)	5,169,712 (13.31%)	33,004,227 (84.96%)
W2 (E13)	W2-1	41,287,160	41,115,972 (99.59%)	97.86%	93.93%	37,117,409 (90.45%)	3,919,621 (9.55%)	36,310,554 (88.48%)
	W2-2	37,696,960	37,541,288 (99.59%)	97.88%	94.01%	33,469,117 (89.33%)	3,996,801 (10.67%)	32,727,856 (87.35%)
	W2-3	41,562,290	41,394,138 (99.60%)	97.91%	94.07%	37,085,128 (89.81%)	4,208,108 (10.19%)	36,280,194 (87.86%)
W3 (E17)	W3-1	41,272,976	41,106,800 (99.60%)	97.53%	93.02%	35,870,494 (87.54%)	5,107,494 (12.46%)	34,856,814 (85.06%)
	W3-2	38,534,114	38,384,120 (99.61%)	97.81%	93.80%	33,301,708 (86.99%)	4,979,908 (13.01%)	32,290,865 (84.35%)
	W3-3	36,793,666	36,656,222 (99.63%)	97.67%	93.31%	31,929,382 (87.36%)	4,621,698 (12.64%)	30,975,611 (84.75%)
W4 (E21)	W4-1	38,136,018	37,985,368 (99.60%)	97.33%	92.68%	31,710,068 (83.75%)	6,154,028 (16.25%)	30,824,726 (81.41%)
	W4-2	36,349,762	36,220,926 (99.65%)	97.88%	93.80%	30,447,014 (84.34%)	5,654,488 (15.66%)	29,627,807 (82.07%)
	W4-3	37,408,416	37,262,530 (99.61%)	97.35%	92.73%	31,054,868 (83.72%)	6,038,858 (16.28%)	30,189,208 (81.39%)
Y1 (E9)	Y1-1	42,228,958	42,064,916 (99.61%)	97.75%	93.61%	37,049,918 (88.25%)	4,930,862 (11.75%)	36,362,036 (86.62%)
	Y1-2	47,208,492	47,009,344 (99.58%)	97.69%	93.51%	42,294,795 (90.16%)	4,615,075 (9.84%)	41,468,940 (88.40%)
	Y1-3	39,561,244	39,397,004 (99.58%)	97.96%	94.16%	35,422,372 (90.13%)	3,877,674 (9.87%)	34,738,884 (88.39%)
Y2 (E13)	Y2-1	37,781,792	37,637,776 (99.62%)	97.86%	93.93%	34,056,615 (90.66%)	3,506,761 (9.34%)	33,334,391 (88.74%)
	Y2-2	39,483,200	39,328,408 (99.61%)	97.97%	94.24%	34,697,347 (88.47%)	4,520,343 (11.53%)	33,951,634 (86.57%)
	Y2-3	36,962,482	36,842,320 (99.67%)	97.80%	93.62%	32,832,856 (89.61%)	3,808,726 (10.39%)	32,144,802 (87.73%)
Y3 (E17)	Y3-1	39,120,498	38,973,346 (99.62%)	97.87%	93.95%	34,524,277 (88.77%)	4,365,509 (11.23%)	33,642,858 (86.51%)
	Y3-2	36,681,408	36,530,244 (99.59%)	97.78%	93.71%	32,225,211 (88.48%)	4,194,471 (11.52%)	31,401,202 (86.22%)
	Y3-3	37,231,224	37,108,648 (99.67%)	97.98%	94.12%	33,275,201 (89.91%)	3,733,489 (10.09%)	32,504,389 (87.83%)
Y4 (E21)	Y4-1	38,291,118	38,126,366 (99.57%)	97.81%	93.85%	31,863,292 (83.90%)	6,116,536 (16.10%)	30,995,200 (81.61%)
	Y4-2	37,590,186	37,455,880 (99.64%)	97.77%	93.59%	30,965,235 (82.97%)	6,357,373 (17.03%)	30,208,722 (80.94%)
	Y4-3	38,522,160	38,363,150 (99.59%)	97.31%	92.61%	32,439,409 (84.86%)	5,786,775 (15.14%)	31,611,797 (82.70%)

Note: W1/2/3/4: Wenchang chickens at embryonic days 9, 13, 17, and 21, respectively. Y1/2/3/4: Yugan chickens at embryonic days 9, 13, 17, and 21, respectively. E9: embryonic day 9; E13: embryonic day 13; E17: embryonic day 17; E21: embryonic day 21.

**Table 2:** Correlation analysis between gene expression and L* value of breast muscle blackness in different breeds of black-boned chickens.

Breeds	MSTRG.720	EDNRB2	DCT	EDN3	TYR	RAB38	OCA2	GPNMB	TRPM1	MLPH
Yugan	0.486 *	−0.611 **	−0.518 **	−0.13	−0.453 *	−0.172	−0.370	−0.571 **	−0.391 *	−0.282
Taihe	−0.337	−0.502 **	−0.444 *	−0.562 **	−0.526 **	−0.681 **	−0.591 **	−0.450 *	−0.431 *	−0.555 **

Note: * indicates *p* < 0.05; ** indicates *p* < 0.01.

## Data Availability

The datasets supporting the results of this study are available in the Sequence Read Archive (SRA) repository under accession number, PRJNA827465.

## Supplementary Materials

The following supporting information can be downloaded at https://www.mdpi.com/article/10.3390/ani14182702/s1. Table S1. The specific primers used for qRT-PCR. Table S2. Detailed information on the DEGs between Wenchang and Yugan black-boned chicken muscle groups at different stages of embryonic development. Table S3. Gene Ontology enrichment analysis of the DEGs between Wenchang and Yugan black-boned chicken muscle groups at different stages of embryonic development. Table S4. KEGG enrichment analysis of the DEGs between Wenchang and Yugan black-boned chicken muscle groups at different stages of embryonic development. Table S5. Genes enriched in the orange module. Table S6. Gene Ontology enrichment analysis of genes in the orange module. Table S7. KEGG pathway enrichment analysis of genes in the orange module. Table S8. Correlation analysis between expression of *MSTRG.720* and other genes in different breeds of black-boned chickens. Table S9. The L* values of breast muscle in different breeds of chickens at 17 weeks of age. Figure S1. Regression analysis of the log 2 (foldchange, FC) values between RNA-seq and qPCR; Figure S2. Morphological photographs of the pectoral muscles of Taihe black-boned chickens at 2 (2 W), 6 (6 W), and 17 weeks (17 W) of age after hatching; Figure S3. The variation patterns of L* value in Yugan (YG) and Taihe (TH) black-boned chicken muscle groups at different developmental stages; Data with different letters on each bar mean statistically significant among different time points for the same breeds (*p* < 0.05); Figure S4. Morphological photographs of the pectoral muscles of Wenchang chickens at 17 weeks age.
