# Supplementary figures and images for: Dynamic Transcriptome Profile Analysis of Mechanisms Related to Melanin Deposition in Chicken Muscle Development
Source: Animals (Basel). 2024 Sep 18;14(18):2702. doi: 10.3390/ani14182702 (PMC11428610; doi:10.3390/ani14182702)

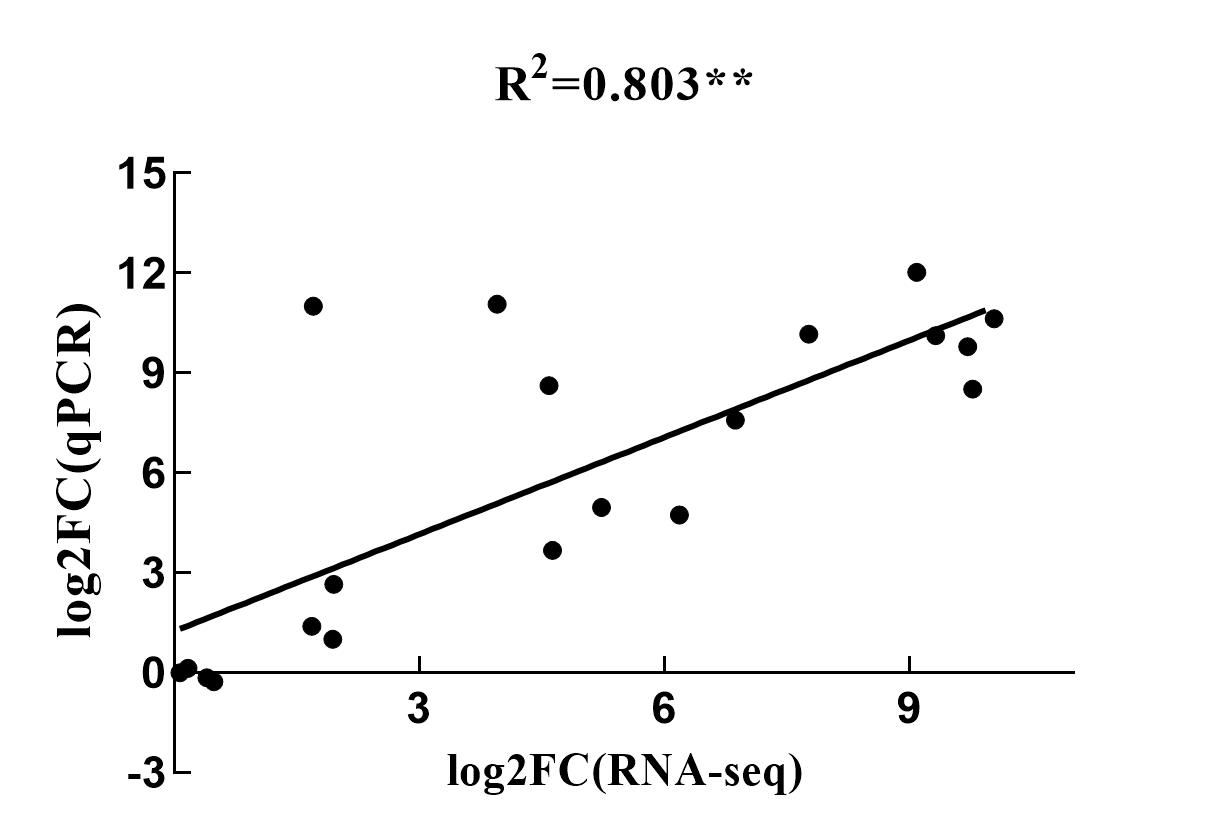

Supplement: Supplementary file 1 [file animals-14-02702-s001.zip › supplementary files/FigureS1.jpg]

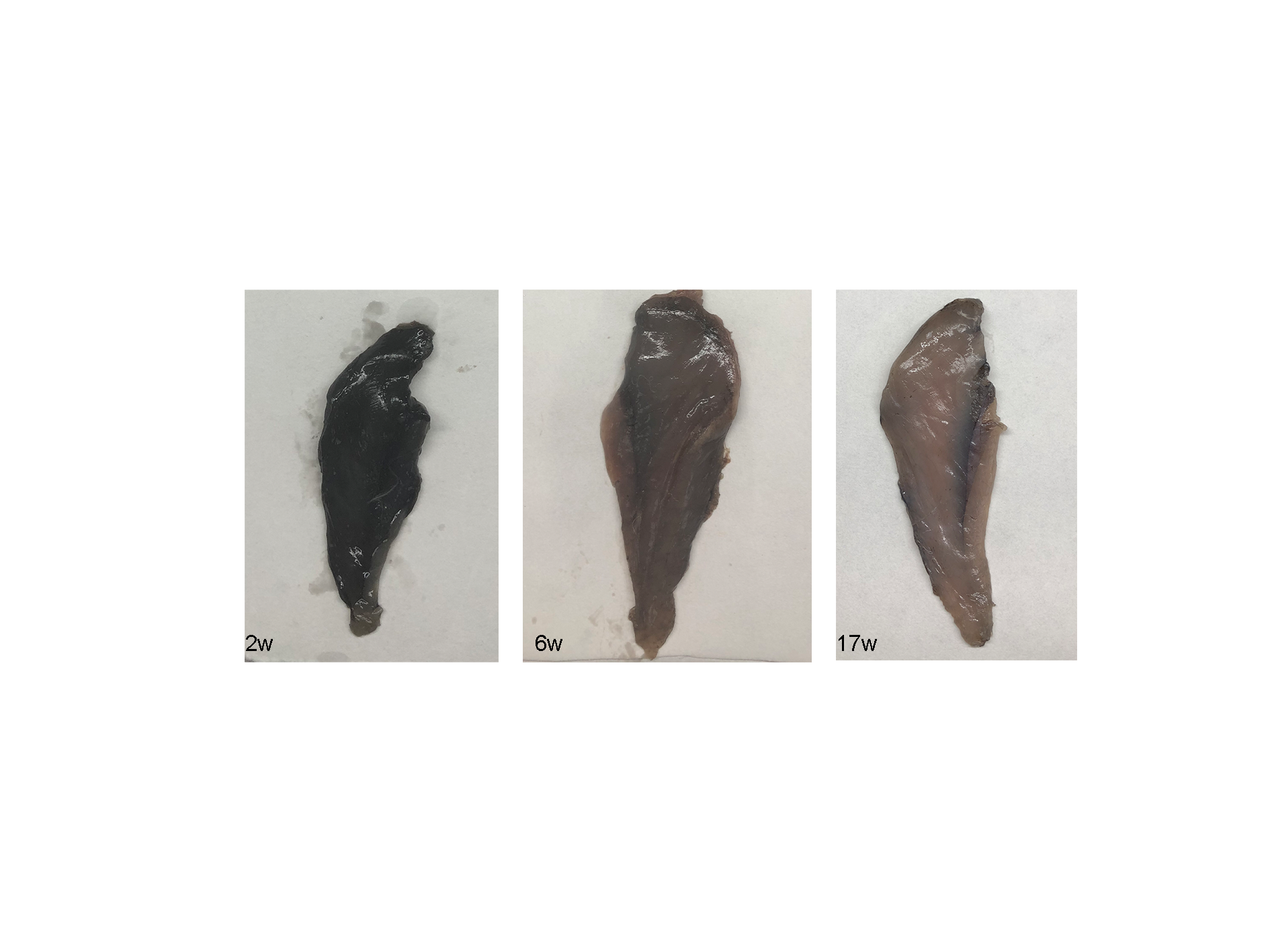

Supplement: Supplementary file 1 [file animals-14-02702-s001.zip › supplementary files/FigureS2.tif]

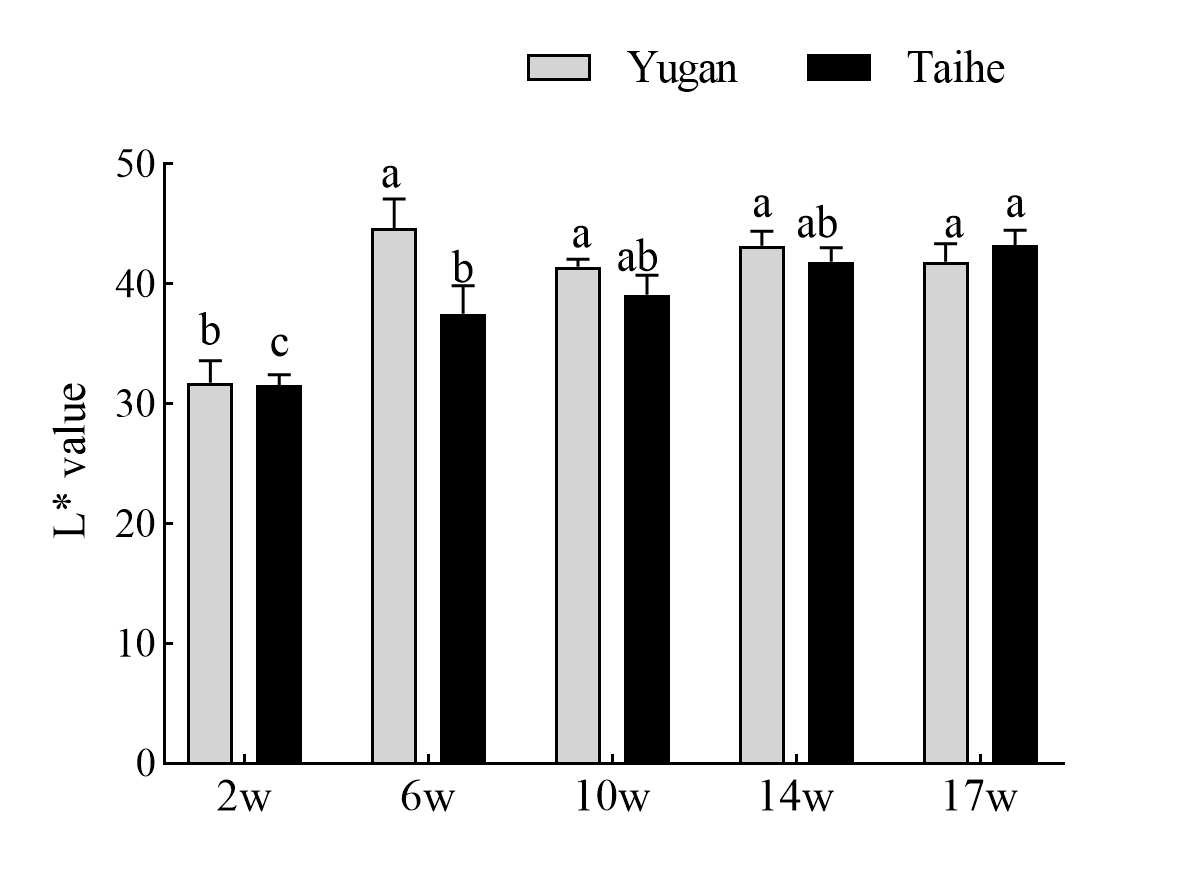

Supplement: Supplementary file 1 [file animals-14-02702-s001.zip › supplementary files/FigureS3.tif]

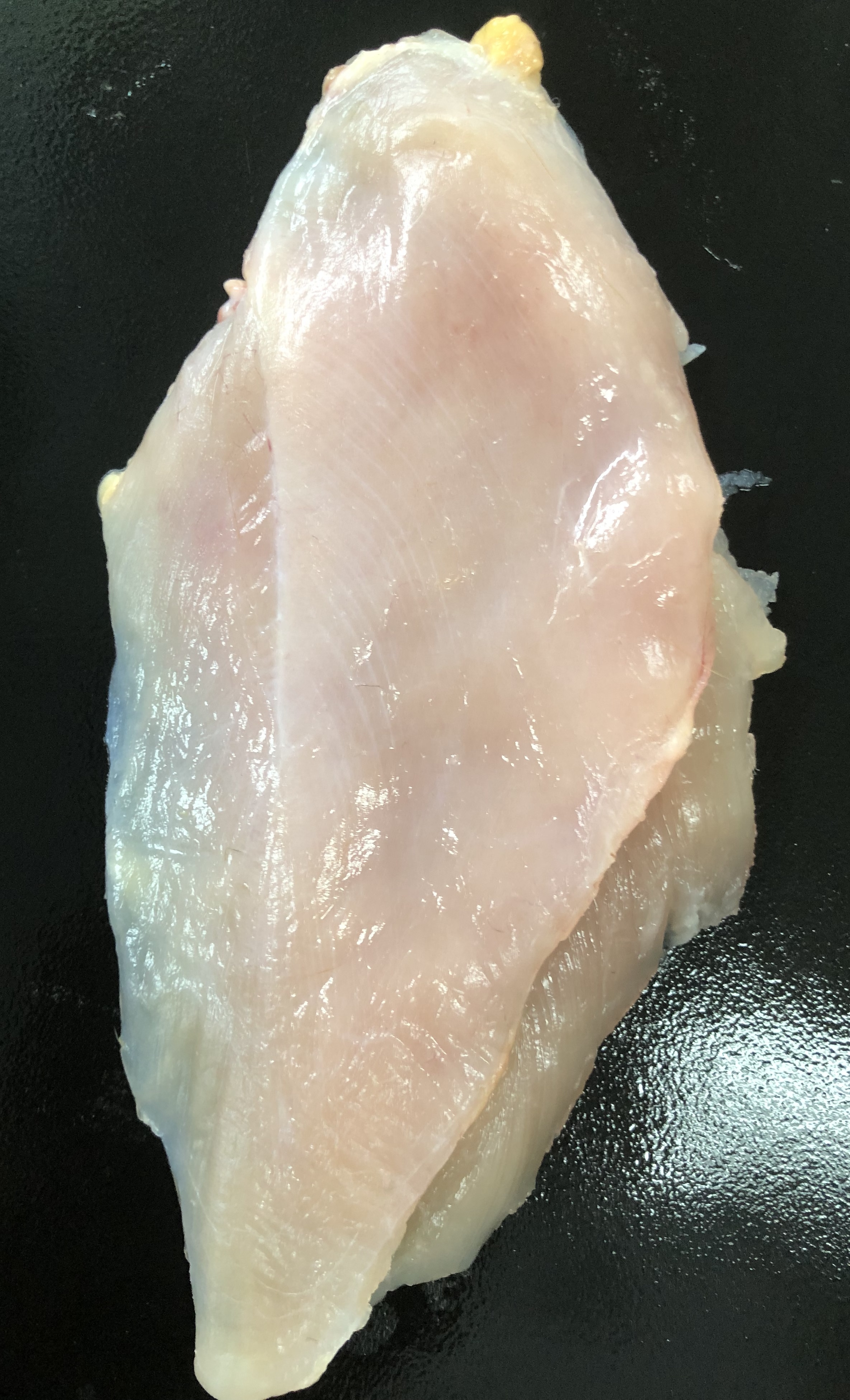

Supplement: Supplementary file 1 [file animals-14-02702-s001.zip › supplementary files/FigureS4.jpg]
